# Reducing Threading Dislocations of Single-Crystal Diamond via In Situ Tungsten Incorporation

**DOI:** 10.3390/ma15020444

**Published:** 2022-01-07

**Authors:** Ruozheng Wang, Fang Lin, Gang Niu, Jianing Su, Xiuliang Yan, Qiang Wei, Wei Wang, Kaiyue Wang, Cui Yu, Hong-Xing Wang

**Affiliations:** 1 Ministry Education Key Laboratory of Physical Electronics and Devices, School of Electronic Science and Engineering, Xi’an Jiaotong University, Xi’an 710049, China; wangrz@xjtu.edu.cn (R.W.); leaf-lin@xjtu.edu.cn (F.L.); jianing1101@stu.xjtu.edu.cn (J.S.); yxl351192351@stu.xjtu.edu.cn (X.Y.); wbgwei@mail.xjtu.edu.cn (Q.W.); wei_wang2014@xjtu.edu.cn (W.W.); 2 Key Laboratory of the Ministry of Education, International Center for Dielectric Research, School of Electronic Science and Engineering, Xi’an Jiaotong University, Xi’an 710049, China; 3 School of Materials Science & Engineering, Taiyuan University of Science & Technology, Taiyuan 030024, China; wangkaiyue8@163.com; 4 National Key Laboratory of Application Specific Integrated Circuit, Hebei Semiconductor Research Institute, Shijiazhuang 050051, China; yucui1@163.com

**Keywords:** tungsten-incorporated diamond, dislocations, XRD, Raman spectroscopy

## Abstract

A lower dislocation density substrate is essential for realizing high performance in single-crystal diamond electronic devices. The in-situ tungsten-incorporated homoepitaxial diamond by introducing tungsten hexacarbonyl has been proposed. A 3 × 3 × 0.5 mm^3^ high-pressure, high-temperature (001) diamond substrate was cut into four pieces with controlled experiments. The deposition of tungsten-incorporated diamond changed the atomic arrangement of the original diamond defects so that the propagation of internal dislocations could be inhibited. The SEM images showed that the etching pits density was significantly decreased from 2.8 × 10^5^ cm^−2^ to 2.5 × 10^3^ cm^−2^. The reduction of XRD and Raman spectroscopy FWHM proved that the double-layer tungsten-incorporated diamond has a significant effect on improving the crystal quality of diamond bulk. These results show the evident impact of in situ tungsten-incorporated growth on improving crystal quality and inhibiting the dislocations propagation of homoepitaxial diamond, which is of importance for high-quality diamond growth.

## 1. Introduction

In the past 10–20 years, the progress of semiconductor materials has created many updated science technologies, such as 5G communication, artificial intelligence, quantum information, and power devices. Of all the semiconductor materials, diamond possesses a wide band gap (5.5 eV), high thermal conductivity (2200 W/mK), high breakdown voltage (10 MV/cm), high carriers’ mobility, high saturation velocity of holes (1.5 × 10^7^ cm/s), and high Johnson’s and Baliga’s figure of merit, making it a promising semiconductor material for next-generation devices [1,2,3,4,5,6,7,8]. However, the existence of defects located in diamond substrates leads to a reduction in the material properties. Defects in diamond include point defects [9], stacking faults (SFs) [10], and threading dislocations (TDs) [11]. Point defects are mainly contained in nitrogen-vacancy centers (NV^0^, NV^−^) [12], silicon vacancy centers (SiV^−^) [13], and hydrogen-related defects [14]. Owing to mechanical damage, SFs usually start from the substrate and then penetrate through the growth layer [15]. It can be removed by chemical mechanical polishing [16]. The dislocation density quantitatively shows the number of defects per unit area. For Ⅱa natural diamond crystal, the dislocation density is 10^8^–10^9^ cm^−2^, which is three to four orders higher than that of synthetic homoepitaxial diamond (10^4^–10^6^ cm^−2^) [17]. Compared to the SFs, the threading dislocations are the main reason for the deterioration of electronic devices performance (i.e., leakage current and breakdown voltage) based on single crystal diamond, especially the dislocations-induced non-linear charge transport mechanisms [18]. Numerous studies have focused on reducing diamond dislocation density. Naamoun et al. revealed a selective masking strategy aiming at preventing threading dislocations propagation by Pt particles. Then, chemical vapor deposition diamond was carried out after a metallic mask [19]. Repeating this procedure several times could lead to a large decrease in the dislocations. Another way to decrease the dislocation density in single crystal diamond is epitaxial lateral overgrowth (ELO). Tallaire et al. proposed a diamond ELO with a large macroscopic hole. The dislocation density in the center was calculated to be approximately 2 × 10^3^ cm^−2^ [20]. Li et al. used a two-step ELO process on an Ib diamond (100) substrate, which combined inductively coupled plasma (ICP) etching and the sputtering of Mo/Pd stripes. The etching pit density was an order of magnitude lower in the two-step ELO layer [21]. Tang et al. proposed a method for high-quality diamond by heteroepitaxial lateral overgrowth. With Au stripe masks on the diamond surface, the stress decreased significantly, and the dislocation density was below 10^8^ cm^−2^ [22]. Kim et al. fabricated a one-inch free-standing heteroepitaxial (001) diamond with a dislocation density of 1.4 × 10^7^ cm^−2^ [23]. Mehmel et al. used micrometric laser-pierced hole arrays to reduce dislocation densities [24]. For diamond thick layer growth, Tsubouchi et al. investigated the microstructures of grown-in threading dislocation bundles in four different growth directions using cross-sectional TEM [25]. Achard et al. found that thick diamond grown on a pyramidal-shaped substrate can lead to dislocation bending and further studied the growth of thick crystals with low dislocation density [9,26]. However, the process of current research is complicated and requires several epitaxial growths, photolithography, sputtering, and etching, and the dislocation density can only be reduced by one to two orders of magnitude. Ohmagari et al. researched homoepitaxial diamond film growth by hot-filament chemical vapor deposition (HFCVD) accompanying tungsten (W) incorporations from heated metal wires. A large reduction in threading dislocation from 2 × 10^6^ to 3 × 10^4^ cm^−2^ was demonstrated by W impurities [27]. However, the diamond films were fabricated in two independent deposition systems, which may lead to new impurities during the growth interval.

In this work, a tungsten-incorporated single-crystal diamond multilayer structure, which could effectively reduce dislocation propagation, was fabricated. At the beginning of the diamond growth, a tungsten precursor was introduced to form a W-C atom-level connection. Then, the tungsten precursor was turned off, and the homoepitaxial diamond continued to grow. The combination of W and C atoms at defects was used to change the arrangement of C atoms at defects, so that C atoms could return to the diamond lattice position, and the propagation of defects (especially dislocations) to diamond films could be inhibited, thereby providing an effective method to reduce the dislocations of homoepitaxial diamond. Compared to the previous studies, the advantage introduced in this work is that the incorporation of W atoms occurs in situ (i.e., in the same deposition chamber) and does not require additional process steps, which would degrade the film quality. This study may put forward a valuable method for growing high-quality and low-defect density single crystal diamond by inhibiting the dislocations propagation, which could be applied in diamond power devices.

## 2. Experimental

The homoepitaxial growth of tungsten-incorporated diamond was fabricated by microwave plasma chemical vapor deposition (MPCVD, AX5250S Seki Technotron Corp. Koto, Japan) on (100)-oriented synthetic high-pressure high-temperature (HPHT) substrate (3 × 3 × 0.5 mm^3^) with the offset angle less than 2°. The substrate was cut into four 1.5 × 1.5 × 0.5 mm^3^ crystals for comparative experiment. The precursor was tungsten hexacarbonyl (W(CO)_6_), which was incorporated into the chamber with hydrogen as the carrier gas. The purity of this solid tungsten source was 99.999%, and the heating temperature is 60 °C. The flow rate was 1.5 sccm, and the W/H ratio, CH_4_ concentration (C/H ratio), and N_2_ concentration (N/H ratio) were 0.3%, 6–7%, and 0.001%, respectively. The deposition temperature and power were 1100 °C and 3800 W, respectively, and the chamber pressure was 160 mbar. The thickness of the tungsten-incorporated layer was 3 μm. Next, the intrinsic diamond was grown in an in situ chamber. The O_2_ concentration (O/H ratio) was 0.5–1%, the deposition temperature was 1050 °C, and the C/H, N/H, and chamber pressures were maintained at the same level. After growth, H_2_/O_2_ (500/5 sccm) plasma was applied to the samples to form etching pits. The etching temperature was 1000 °C, and the duration was 30 min.

The dark current was tested by a semiconductor characterization system (Keithley 4200). A 6-circle XRD (SmartLab, Rigaku, Tokyo, Japan) system was used to test the crystallinity of the diamond containing both the substrate and epitaxial layers [28]. The confocal micro-Raman spectrometer (Renishaw inVia, New Mills, UK) was used to evaluate the diamond defect structures. The laser is a 532 nm Nd: sapphire with laser power of 1%, the excitation power is 50 mW, and the resolution of the spectrometer in the horizontal and depth directions is 1 μm. The microstructure and dislocation densities (dislocations crossing unit area) were observed using an SEM (Gemini SEM 500, Zeiss, Dresden, Germany). The concentrations of tungsten and nitrogen impurities were determined using SIMS (Cameca 4F, Gennevilliers, France).

## 3. Results and Discussion

**Figure 1:**
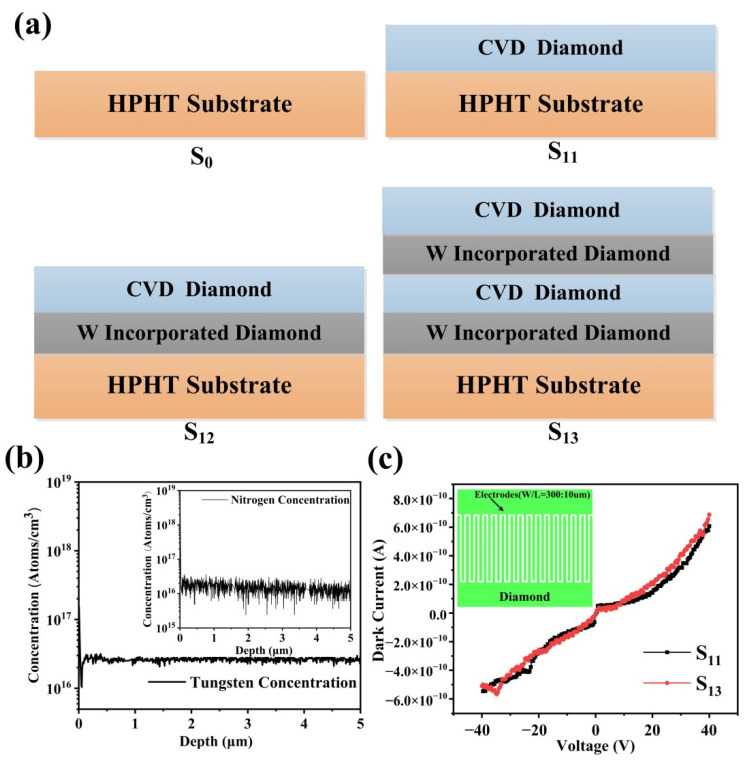
(**a**). Cross-section structures of substrate (S_0_) and three other epitaxial diamond structures. (S_11_, S_12_, and S_13_); (**b**). W concentration in tungsten-incorporated diamond tested by SIMS (inset is the nitrogen concentration). (**c**) I–V curves of S_11_ and S_13_ under dark condition.

The variation of film structures is shown in Figure 1a. S_0_ was the HPHT substrate, which was set as a controlled sample. For S_11_, a 10 μm intrinsic diamond was grown on the substrate. Single and double layers of tungsten-incorporated diamond (3 μm) were deposited for S_12_ and S_13_, respectively. The W concentration was measured by SIMS with another controlled sample (10 μm), which was shown in Figure 1b. The W concentration was uniform in diamond bulks, showing the good consistency of approximately 2 × 10^16^/cm^3^ in the whole film, which was two orders of magnitude lower than the present work (10^18^/cm^3^) [27].

Meanwhile, the nitrogen concentration was close to the 10^16^ cm^−3^ (shown in the inset of Figure 1b), which could come from unintentional nitrogen in the chamber. Furthermore, in order to explore the carrier’s transportation characteristics of tungsten-incorporated diamond, the I–V characteristics of intrinsic diamond (S_11_) and double-layer tungsten-incorporated diamond (S_13_) under dark condition are shown in Figure 1c. It was obvious that the dark currents of the two samples almost maintain consistency. When the voltage was applied at 40V, the relatively low dark current of S_11_ and S_13_ are 6.07 × 10^−10^ A and 6.87 × 10^−10^ A, respectively, indicating that the carrier’s transportation was not changed with the introduction of tungsten-incorporated diamond. The N concentration in tungsten incorporated diamond was also tested by SIMS (shown in supplementary Figure S1), and the average value was close to 10^16^ cm^–3^. 

**Figure 2:**
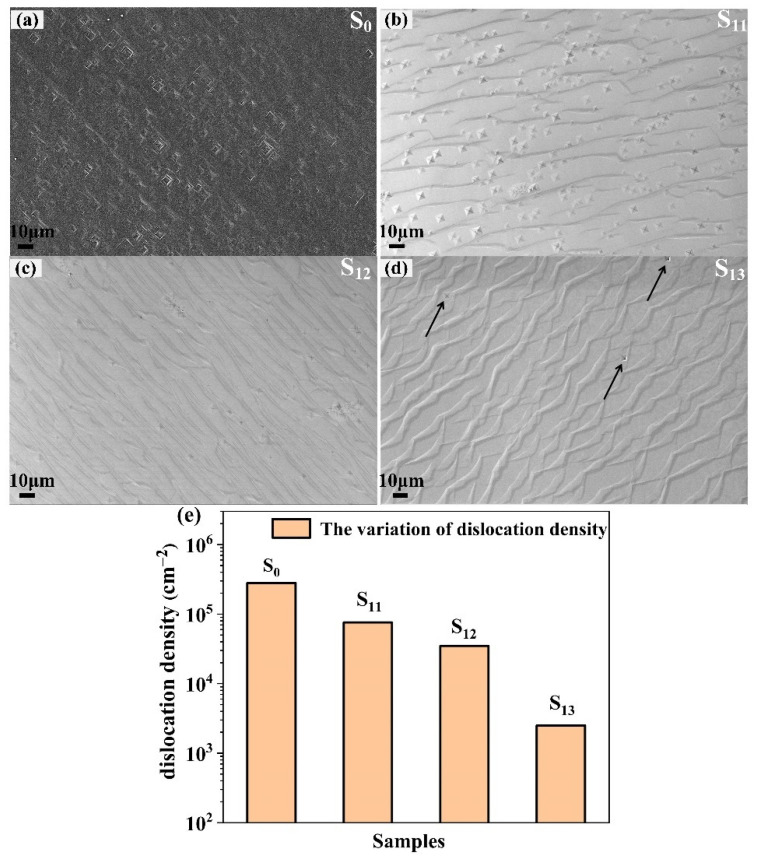
SEM images of four etched samples. (**a**) S_0_; (**b**) S_11_; (**c**) S_12_; (**d**) S_13_; (**e**) the variation of dislocation density in four samples.

The effect of tungsten-incorporated diamond layer on the dislocation reduction could be revealed directly from the SEM images after H_2_/O_2_ etching. Figure 2a–d showed the morphology of the four samples under the same magnification, and the square-pits along (110) directions can be originated from dislocations [19]. Compared to the samples (S_11_, S_12_, and S_13_) deposited in MPCVD, the etching pits density of S_0_ was the highest, indicating that the HPHT diamond substrate contained many defects. To be specific, Figure 2b–d showed the step flow morphology of homoepitaxial diamond [29]. The trend of dislocation density is shown in Figure 2e. The dislocation density of S_0_ and S_11_ stayed at the same level (2.8 × 10^5^ cm^−2^ and 7.6 × 10^4^ cm^−2^, respectively), which meant that the intrinsic diamond layer could not effectively stop the propagation of dislocations. Furthermore, the etching pits densities were reduced significantly in S_12_ and S_13_. Particularly, only three pits were observed in S_13_, and the dislocation densities were 3.5 × 10^4^ cm^−2^ and 2.5 × 10^3^ cm^−2^, respectively, which proved an effective method of reducing dislocation density.

**Figure 3:**
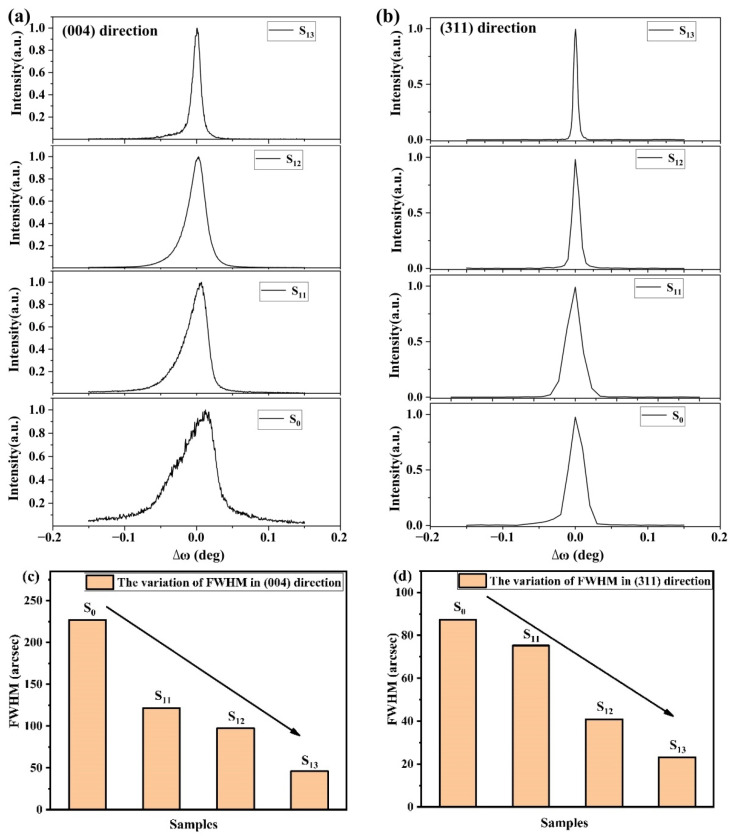
XRD pattern of ω scan in diamond. (**a**) (004) reflection; (**b**) (311) reflection; (**c**) the variation of FWHM in (004); (**d**) the variation of FWHM in (311).

Furthermore, tungsten incorporation will also improve the crystal quality of diamond. The crystallinity of the four samples is shown in Figure 3, and the ω scans were obtained in the diamond (004) symmetric diffraction and (311) asymmetric diffraction, which represented the decent single crystal characteristics. In Figure 3a, stronger asymmetric broader peaks (S_0_ and S_11_) have been detected, which are attributed to the defects in the diamond lattice. Meanwhile, the well-performed XRD rocking curves of S_12_ and S_13_ with the narrower peak corresponded to tungsten-incorporated diamond. The full widths at half maximum (FWHM) in (004) and (311) reflections (shown in Figure 3c,d) of S_0_ extracted from the ω-scan were 226.8 and 87.3 arcsec, representing the worse crystallinity of the HPHT substrate [30,31]. Then, the diffraction peaks of S_11_ to S_13_ were narrowed after intrinsic diamond and tungsten-incorporated diamond deposition, and the FWHM (004) of S_11_ to S_13_ were 121.3, 97.2, and 46.0 arcsec respectively. While the FWHM (311) of S_11_ to S_13_ were 75.24, 40.89, and 23.22 arcsec respectively. As we mentioned before, the substrates were cut from the same 3 × 3 mm^2^ HPHT substrate for controlled experiments, indicating that the crystallinity of the whole diamond bulk was improved by tungsten-incorporated diamond.

**Figure 4:**
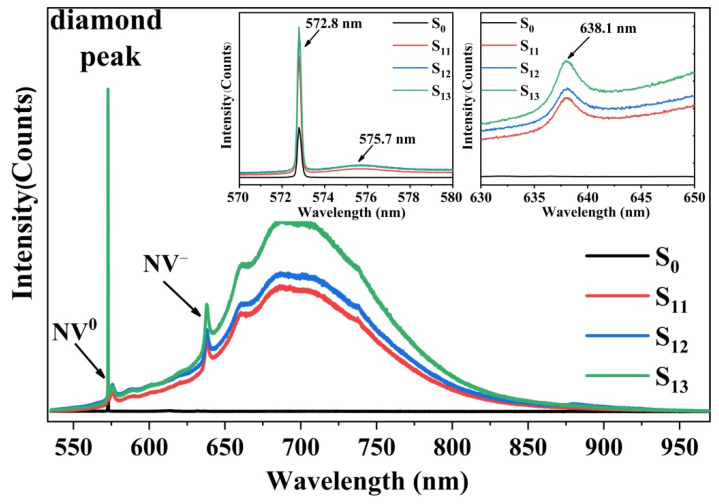
Normalized PL spectra of S_0_, S_11_, S_12_, and S_13_. (The insets show the wavelength ranges of 570–580 and 630–640 nm).

Additionally, in order to evaluate the origin of defects in tungsten-incorporated diamond, the research of photoluminescence (PL) spectra was carried out as well as an investigation of correlation of the defect to Raman spectroscopy peak width and shift. Figure 4 showed the normalized intensity of PL spectra among four combined samples. The insets showed the amplification of the 570–580 nm and 630–650 nm wavelength. The resolution of the spectrometer in the horizontal and depth directions was 1 μm, so that the PL and Raman measurements were made for individual layers. The sharp Raman line at 572.8 nm indicated the high quality of diamond. There were two relatively strong zero phonon lines (ZPL) at 575.7 nm and 638.1 nm, which represented NV^0^ and NV^−^ [32]. Before growth, there was no NV center in the substrate. The obvious NV^0^ and NV^−^ characteristic peaks were detected after homoepitaxial diamond deposition (S_11_ to S_13_), illustrating that more vacancies were introduced during tungsten-incorporated diamond growth. The possible reasons were as follows: under high-temperature and plasma conditions, the C atoms in the diamond lattice were partially separated from the original lattice and reacted with W atoms to form vacancies. Then, NV centers in diamond (nitrogen concentration was about 10^16^ cm^−3^, which was observed in the inset of Figure 1b) were formed with the movement of vacancies at high temperatures [12]. Furthermore, NV centers were stronger in the S_12_ and S_13_ samples, which meant that the TID layer could effectively enhance the density of NV centers, especially for NV^−^ [33].

**Figure 5:**
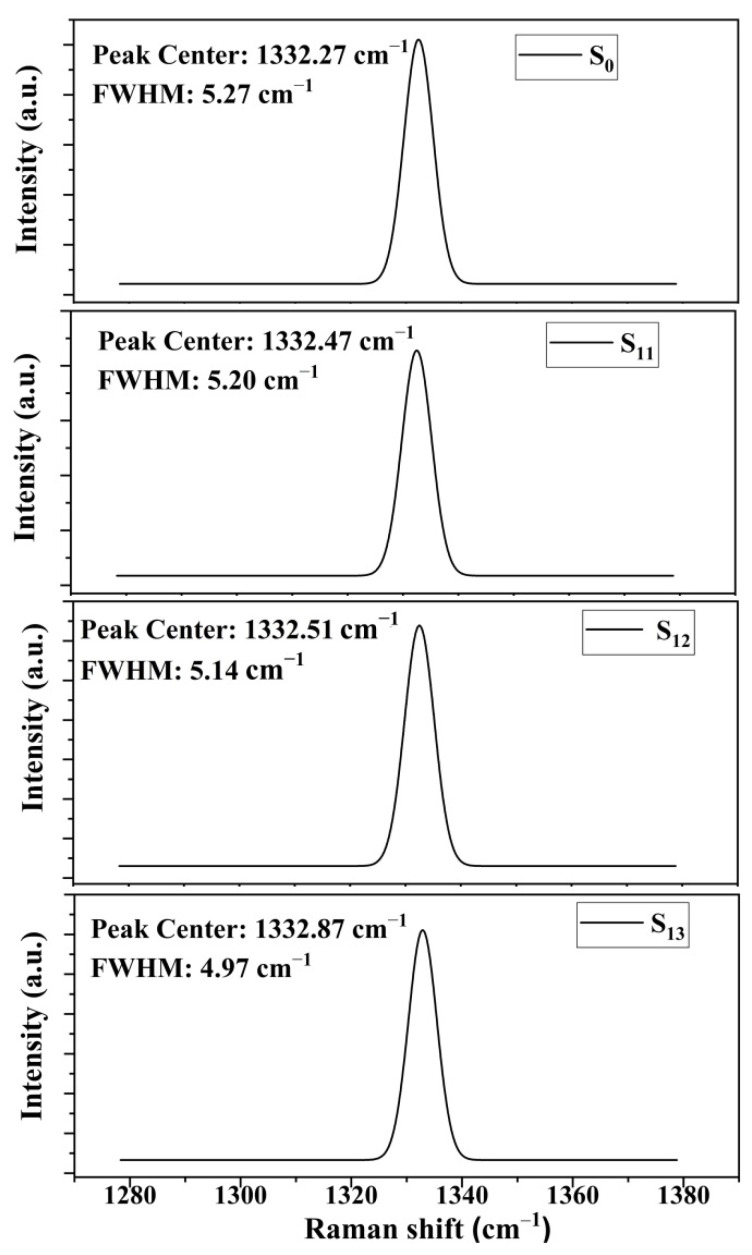
The variation of Raman spectroscopy extracted from S_0_, S_11_, S_12_, and S_13_.

To further discussion, the variation of Raman spectroscopy with four samples are shown in Figure 5. All images showed the characteristics peaks of diamond SP^3^ orbital hybridization. Overall, we could see clearly that the peak of S_0_ showed almost consistency with intrinsic diamond (1332 cm^−1^). However, the shift of peaks that occurred in S_11_, S_12_, and S_13_ reached 0.47, 0.51, and 0.9 cm^−1^, respectively, which could probably be caused by nitrogen addition. The research by Bergman et al. illustrated that the positive shift in Raman peak was attributed to the nitrogen-induced expansion stress [34]. As mentioned in Figure 4, the intensity of NV centers (NV^0^ and NV^−^) are strongest in S_13_, which is in correlation with the largest shift of Raman peak. In addition, it is inferred that with the incorporation of W, the recombination of W and C atoms will produce new vacancies, which probably lead to the shift of Raman peak as well. Furthermore, the variation of FWHM in Raman spectroscopy was usually used to characterize the crystal quality in the diamond; i.e., a narrow FWHM corresponded to better crystallinity and lower defects density [35], which was also mentioned in the explanations of Figure 5. The FWHM values of S_0_, S_11_, S_12_, and S_13_ are 5.27, 5.20, 5.14, and 4.97 cm^−1^, respectively. As we have already known, the line width of Raman was closely related to the defects in diamond lattice. The nitrogen defects were extensive in the HPHT substrate, which would disturb the crystal lattice and induce infrared absorption in the one-phonon region. Then, the mean free path of phonons became shorter as they experienced additional scattering on nitrogen defects, leading to the widening of Raman peaks [36]. Therefore, the FWHM of S_0_ was the largest. After the MPCVD growth of intrinsic diamond and a tungsten-incorporated diamond layer, the narrower of line width in S_11_ to S_13_ could probably be attributed to the following reasons: the reduction of dislocation density by introducing the tungsten-incorporated diamond layer, and the probability of a phonon scattering on nitrogen defects was stronger than zero phonon lines (NV^0^ and NV^−^). 

## 4. Conclusions

In this paper, a low dislocation density tungsten-incorporated diamond growth by in situ microwave plasma chemical vapor deposition has been proposed. The tungsten hexacarbonyl is introduced into the reaction chamber with hydrogen as a carrier gas. As a result, tungsten atoms replace the defects in the diamond lattice and repair the lattice mismatch at the defects, inhibiting the propagation of dislocations. The dislocation densities of the tungsten-incorporated diamond were reduced by two orders of magnitude (2.8 × 10^5^ cm^−2^ vs. 2.5 × 10^3^ cm^−2^). As a result of the lower concentration of tungsten (2 × 10^16^ cm^−3^), the carrier transport characteristics of diamond do not change by testing the I–V characteristics of S_11_ and S_13_ under dark condition. The FWHM of the XRD ω-scan is 23.22 arcsec in the (311) direction, indicating that the crystal quality of tungsten-incorporated diamond could reach the level of synthetic Ib diamond. This study provides a simplified in situ method for high-quality, low-defect density homoepitaxial diamond growth, which is of great importance in diamond electronic devices. Further studies could be extended to the tungsten incorporation to control the concentration, electron spin, and fluorescence lifetime of NV centers in diamond, which makes diamond NV centers the potential applications in quantum information technology.

## Data Availability

Data available on request due to restrictions, e.g., privacy or ethical.

## Supplementary Materials

The following supporting information can be downloaded at: https://www.mdpi.com/article/10.3390/ma15020444/s1, Figure S1. N concentration in TID tested by SIMS.
