# Supplementary material for: Reducing Threading Dislocations of Single-Crystal Diamond via In Situ Tungsten Incorporation
Source: Materials (Basel). 2022 Jan 7;15(2):444. doi: 10.3390/ma15020444 (PMC8778734; doi:10.3390/ma15020444)
Supplement: Supplementary file 1 [file materials-15-00444-s001.zip › materials-1491063-supplementary.pdf]

materials

Supplementary Material

# Reducing Threading Dislocations of Single-Crystal Diamond via In Situ Tungsten Incorporation

**Ruozheng Wang [1], Fang Lin [1], Gang Niu [2,*], Jianing Su [1], Xiuliang Yan [1], Qiang Wei [1], Wei Wang [1], Kaiyue Wang [3], Cui Yu [4] and Hong-Xing Wang [1,*]**

[1] Ministry Education Key Laboratory of Physical Electronics and Devices, School of Electronic Science and Engineering, Xi'an Jiaotong University, Xi'an 710049, China; wangrz@xjtu.edu.cn (R.W.); leaf-lin@xjtu.edu.cn (F.L.); jianing1101@stu.xjtu.edu.cn (J.S.); yxl351192351@stu.xjtu.edu.cn (X.Y.); wbgwei@mail.xjtu.edu.cn (Q.W.); wei_wang2014@xjtu.edu.cn (W.W.)
[2] Key Laboratory of the Ministry of Education & International Center for Dielectric Research, School of Electronic Science and Engineering, Xi'an Jiaotong University, Xi'an 710049, China
[3] School of Materials Science & Engineering, Taiyuan University of Science & Technology, Taiyuan 030024, China; wangkaiyue8@163.com
[4] National Key Laboratory of Application Specific Integrated Circuit, Hebei Semiconductor Research Institute, Shijiazhuang 050051, China; yucui1@163.com
* Correspondence: gangniu@xjtu.edu.cn (G.N.); hxwangcn@mail.xjtu.edu.cn (H.-X.W.)

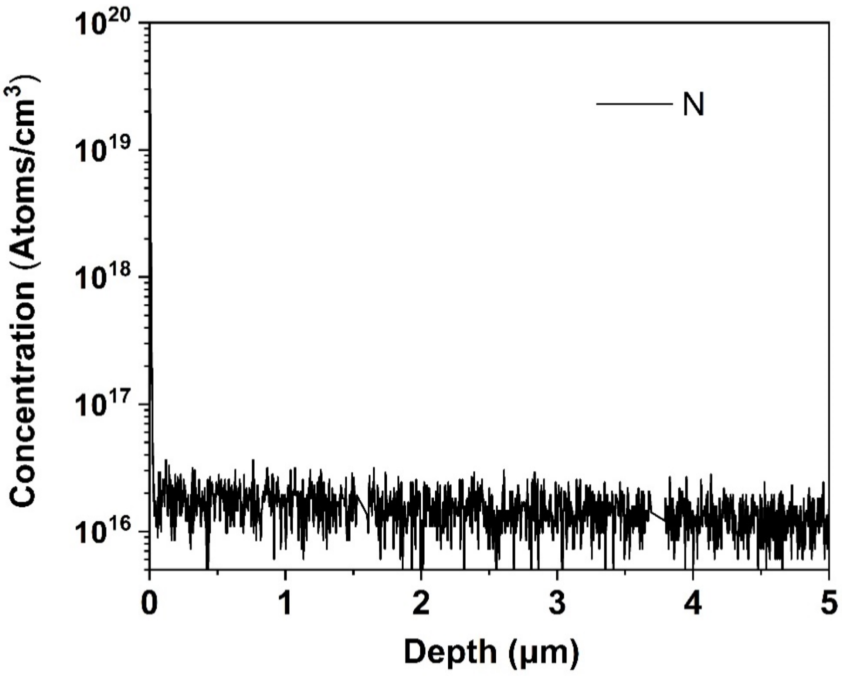


**Figure S1.** N concentration in TID tested by SIMS.
